# Oncolytic parvoviruses: from basic virology to clinical applications

**DOI:** 10.1186/s12985-014-0223-y

**Published:** 2015-01-29

**Authors:** Antonio Marchini, Serena Bonifati, Eleanor M Scott, Assia L Angelova, Jean Rommelaere

**Affiliations:** Infection and Cancer Program, Tumor Virology Division (F010), German Cancer Research Center (DKFZ), Im Neuenheimer Feld 242, 69120 Heidelberg, Germany

**Keywords:** Oncolytic parvoviruses, Parvovirus-based cancer virotherapy, Parvovirus-based oncolytic vectors, Parvovirus-based combination therapy, *Rodent protoparvovirus*, Glioblastoma multiforme, Oncolytic virotherapy clinical trial

## Abstract

Accumulated evidence gathered over recent decades demonstrated that some members of the *Parvoviridae* family, in particular the *rodent protoparvoviruses* H-1PV, the minute virus of mice and LuIII have natural anticancer activity while being nonpathogenic to humans. These studies have laid the foundations for the launch of a first phase I/IIa clinical trial, in which the rat H-1 parvovirus is presently undergoing evaluation for its safety and first signs of efficacy in patients with glioblastoma multiforme. After a brief overview of the biology of parvoviruses, this review focuses on the studies which unraveled the antineoplastic properties of these agents and supported their clinical use as anticancer therapeutics. Furthermore, the development of novel parvovirus-based anticancer strategies with enhanced specificity and efficacy is discussed, in particular the development of second and third generation vectors and the combination of parvoviruses with other anticancer agents. Lastly, we address the key challenges that remain towards a more rational and efficient use of oncolytic parvoviruses in clinical settings, and discuss how a better understanding of the virus life-cycle and of the cellular factors involved in virus infection, replication and cytotoxicity may promote the further development of parvovirus-based anticancer therapies, open new prospects for treatment and hopefully improve clinical outcome.

## Background

**Figure 1:**
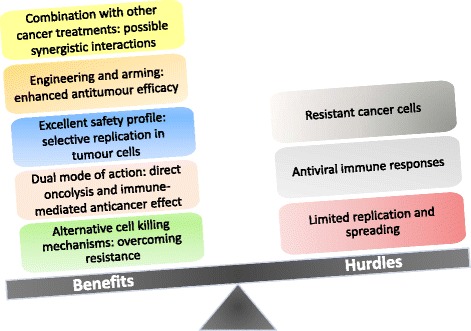
**Pros and cons of oncolytic virotherapy.** Illustrated are the main advantages of oncolytic virotherapy and the major challenges that remain to be tackled in order to improve clinical outcome.

The idea to use viruses as tools for cancer therapy arose as early as at the turn of the 20th century, when it was reported that leukemia patients who contracted influenza went into clinical remission. Although anecdotal, these observations prompted intensive investigation of treatment strategies based on viruses with inherent anticancer activity, leading to the launch of the first clinical trials in the 1950s and 60s. Despite early promise, concerns regarding safety and a lack of efficacy caused a diminishment in oncolytic virotherapy research during the years that followed. However, with dramatic advances in our understanding of molecular biology and virology and the advent of genetic engineering, the last two decades have brought with them a resurgence of interest in the field (for a review on the history of oncolytic virotherapy see [1]). Virotherapeutics can be subdivided into two groups: (i) replication-deficient virus vectors, which are used to deliver therapeutic genes to the target tumor, and (ii) replication-competent oncolytic viruses (OVs). The latter possess the ability to selectively infect, replicate in and destroy tumor cells, while sparing their normal counterparts. In addition to their direct oncolytic effect, OVs induce antitumor immune responses. Importantly, the balance between antitumor and antiviral immune reactions plays a major role in the efficiency of OV-mediated tumor suppression. Figure 1 summarizes major advantages and limitations of oncolytic virotherapy. For a general introduction to the field we redirect the readers to these excellent recent reviews [2,3]. As a result of their oncosuppressive abilities demonstrated in numerous preclinical models, no fewer than twelve OVs are currently being evaluated in clinical trials against a number of different cancers. In particular, a modified herpes simplex virus (HSV) (OncoVEX GM-CSF) [4] and reovirus (Reosyn, Reolysin) [5] have reached phase III clinical trials against recurrent melanoma and head and neck cancer, respectively, and a recombinant vaccinia virus (JX-594, Pexa-Vec) is currently undergoing evaluation in a randomized phase IIb clinical study against recurrent hepatocellular carcinoma [6]. Results are very promising and there is a justified optimism that these agents may be approved by the regulatory agencies in the near future and become real therapeutic options for cancer patients with these diseases. Among OVs, the Rodent protoparvovirus 1 (RoPV) species within the *Parvoviridae* family deserves special consideration for its promising anticancer properties. The RoPV viruses exert striking oncosuppressive effects in various preclinical tumor models, are able to kill tumor cells which resist conventional treatments, and have not been associated with disease in humans, laying the basis for the launch of the first phase I/IIa clinical trial using the rat oncolytic H-1 parvovirus (H-1PV).

### Basic characteristics of oncolytic parvoviruses

#### Classification

The *Parvoviridae* family presently includes 134 viruses that infect a broad range of hosts. They are characterized by an icosahedral capsid of about 25 nm in diameter containing a linear, single-stranded DNA molecule [7]. The family is divided in two subfamilies, *Parvovirinae* and *Densovirinae*, members of which infect vertebrates and arthropods, respectively [7]. Eight genera have been classified as belonging to the *Parvovirinae* subfamily. The focus of the present review is on one of these genera, *Protoparvovirus*, and more particularly on one of its species, *Rodent protoparvovirus* 1 (RoPV1), whose members are able to replicate autonomously in close dependence on cellular S-phase factors. RoPVs include the H-1 parvovirus (H-1PV), the major subject of this review, the Kilham rat virus (KRV), the LuIII virus, the Mouse parvoviruses (MPV) and the Minute viruses of mice (MVM). In unprotected fetuses and neonates of the natural or related hosts, RoPV infection can be pathogenic and even lethal, whilst in adults the infection is clinically inapparent though persistent. Interestingly, these viruses are able to replicate in cells of different origins, including transformed human cells, as it will be extensively discussed in the next paragraphs.

#### Rodent protoparvovirus structure and products

**Figure 2:**
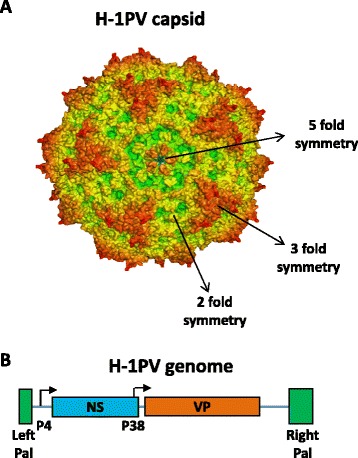
**The oncolytic rat parvovirus H-1PV. A)** A model of the icosahedral capsid is illustrated showing the 2, 3 and 5 fold axes of symmetry [28] **B)** The 5 kb single-stranded linear DNA genome has unique palindromic terminal sequences (Pal) that serve as self-priming origins of replication. Transcription is regulated by the P4 and P38 promoters, whose position is indicated by arrows. There are two transcription units coding for the non-structural (NS) and structural (VP) proteins, respectively.

The RoPV capsid consists of 60 copies of two to three polypeptide sequences represented by the capsid proteins VP1, VP2 and VP3 [8]. The capsid structure is characterized by three main elements: (i) a spike-like protrusion at the 3-fold axis of symmetry; (ii) a depression, called dimple, at the 2-fold axis; (iii) a pore located at the 5-fold axis, connecting the inside of the virion to the exterior [9] (Figure 2A).

The parvoviral genome is a linear, single-stranded DNA molecule of around 5 kb comprising two transcription units that respectively code for non-structural proteins (NS) involved in the replication process and in viral cytotoxicity, and structural proteins (VP) forming the capsid. The expression of viral genes is regulated by two promoters, P4 and P38 [10]. P4 controls the expression of the non-structural proteins NS1, NS2 (and a putative NS3 read-through product). P38 regulates the production of the capsid proteins VP1 and VP2 (and of a minor regulatory protein called SAT) and is transactivated by the NS1 protein. At both termini of the viral DNA, coding sequences are flanked by short terminal palindromes, whose size varies between the left (approx. 120 nt) and the right (approx. 250 nt) terminus, and which are essential for the replication process (Figure 2B).

The parvoviral product NS1 is a multifunctional phosphoprotein of 672 amino acids with a theoretical molecular weight of 83 kDa. It is mainly located in the nucleus due to a nuclear localization signal (NLS) within its sequence [11], although a minor but significant fraction of the protein remains cytoplasmatic. NS1 contains a DNA-binding domain in the N-terminal region [12] and a transcriptional activation domain located at the C-terminus, responsible for the regulation of the P38 promoter and of its own P4 promoter activities [8]. NS1 binds to the viral genome as homodimers or multimers, conformations promoted by ATP.

NS1 plays a major role in the RoPV life cycle, regulating both viral DNA replication and gene expression. It has been shown that NS1 is target for post-translational modifications that regulate protein functions. For instance, phosphorylation on different amino-acid residues catalysed by cellular protein kinases C (PKC), regulates both enzymatic (helicase and ATPase) and non-enzymatic (DNA binding and transcriptional activation) NS1 activities [10]. Furthermore, recent studies have described that, in addition to phosphorylation, acetylation also plays an important role in modulating the cellular activities of the H-1PV NS1 protein and in particular the ability of the protein to bind to DNA, to transactivate the P38 promoter and regulate viral replication [13].

Besides being involved in the regulation of the parvoviral life cycle, as will be discussed in the next paragraphs, NS1 is also the major effector of viral cytotoxicity and its sole expression is sufficient to induce cell cycle arrest and apoptosis [14-16].

The RoPV protein NS2 has a molecular mass of about 25 kDa and a mainly cytoplasmic localization. In the case of MVM and related viruses, alternative splicing produces three different isoforms, NS2Y, NS2L and NS2P. The exact role of NS2 in the viral life cycle and cytotoxicity is still elusive. However, studies performed in mouse cells infected with MVM demonstrated a role of NS2 in the synthesis of DNA replicative forms and progeny production [8]. Furthermore, it has been shown that the interaction of NS2, through a nuclear export sequence, with chromosome region maintenance protein 1 (CRM1) is required for efficient nuclear egress of progeny virions in mouse cells [17].

Recently, a novel non-structural protein has been identified, namely the small alternatively translated (SAT) protein, which is conserved among all members of the *Protoparvovirus* genus [18]. The open reading frame of this protein is located a few nucleotides downstream of the VP2 initiation codon. The protein localizes to the endoplasmic reticulum (ER) in Porcine parvovirus-infected cells and, although its role during infection remains elusive, it has been suggested to block major histocompatibility complex (MHC) type I processing and/or trigger ER stress-induced cell death, similarly to other ER-targeted viral proteins [18].

The structural proteins VP1 and VP2 present the same nucleotide sequence except for the N-terminal domain: VP1 contains a unique sequence of 140 to 230 residues, besides the complete sequence of VP2. These additional residues have been shown to be important for viral infectivity but not for the packaging process [19,20]. The VP1 unique region displays a phospholipase A_2_ (PLA_2_) activity, which is conserved among *Parvovirinae* members and thought to be required for the transfer of viral genomes from the endocytic compartment to the cytosol [21-24]. VP2 is the most abundant protein of the capsid representing 90% of the entire structure. VP3 is generated by post-assembly proteolytic cleavage of 25 amino acids at the N-terminus of VP2 and is found only in small amounts in infectious particles [25].

#### Protoparvovirus entry, replication and egress

Virus entry into target cells occurs through the interaction of the viral capsid with surface cellular receptors, which have been identified only for some members of the *Parvovirinae* subfamily. For instance, the Feline and Canine parvoviruses, belonging to the genus *Protoparvovirus*, require the transferrin receptor for the entry process [26]. Regarding the Rodent protoparvovirus species, sialic acid has been identified as an important mediator for cell membrane recognition and entry for the MVM and H-1PV viruses [27-29], though the cognate cell receptors remain to be identified.

Viral capsids enter target cells through an endocytic mechanism, which is thought to involve clathrin proteins [30], and transports virions to perinuclear regions [25,31,32]. The PLA_2_ activity of the VP1 protein triggers the degradation of the endosome by modification of its membrane composition, thereby favoring the release of viral particles into the cytoplasm [21-24]. The intracellular trafficking of viral particles mainly involves the microtubule network [33] and viral entry into the nucleus occurs through the nuclear pore complex (NPC). The interaction of the virus with proteins of the NPC has been shown to lead to the exposure of the unique sequence of the VP1 viral protein containing nuclear localization signals (NLS) besides the PLA_2_ domain. In some experimental systems, *Protoparvovirus* members have proven able to induce disruption of the nuclear envelope through activation of key enzymes of mitosis [34]. However, this nuclear breakdown process is unlikely to be the normal pathway of viral entry into the nucleus during infection of most cells. *Protoparvovirus* genome replication is strictly dependent on S-phase cellular factors. Therefore, incoming virions remain silent, without integrating their DNA into the cellular genome, until the host cell enters S-phase. As the cell expresses S-phase factors, viral DNA replication initiates. The replication process comprises the conversion of the single-stranded DNA molecule into a double-stranded replicative form. The newly synthesized double-stranded DNA molecules are used as templates for viral mRNA synthesis and for the formation of multimeric double-stranded intermediates and progeny DNA molecules [8]. Viral genome replication occurs in well-defined nuclear structures called “autonomous parvovirus-associated replication (APAR) bodies and requires the recruitment of cellular factors such as replication protein A (RPA), proliferating cell nuclear antigen (PCNA), DNA polymerase α and δ and cyclin A [35,36]. Subsequent activation of the P4 promoter and expression of NS1 and NS2 proteins rely on the cellular transcription factors E2F, ATF/CREB, ETS and NF-Y which are overexpressed in fast replicating cells [10,37]. These various factors mediate at least in part the RoPV dependence on cellular proliferation and transformation. MVM and H-1PV infection is associated with the induction of a DNA damage response (DDR) and consequent cell cycle arrest (see below) [16,38]. It has been proposed that virus-induced cell cycle arrest perpetuates conditions favorable for virus replication [16,39]. NS1, in turn, activates the P38 viral promoter, inducing the expression of VP capsid proteins, and guides the DNA replication and encapsidation processes. These steps of the parvoviral life cycle involve the direct interaction of NS1 with various cellular factors, including RPA and the transcription factors TBP, TFIIA and SP1/3 [10]. This virus-cell interplay ensures the progression of viral replication and gene expression on the one hand, and disturbs cellular metabolic processes on the other hand [10].

After the expression of the capsid proteins, new virions are produced and released, most likely through an active mechanism which brings the progeny virions from the nucleus to the cell periphery. Indeed, it has recently been reported that RoPV egress from the host cell occurs through the formation of virus-containing cellular vesicles in the endoplasmic reticulum, which are then transported to the membrane through the Golgi in a gelsolin-dependent manner [40]. Transport from the endoplasmic reticulum to the Golgi is mediated by cellular proteins such as moesin and radixin and seems to be an important step in virus infection, during which parvoviral particles undergo post-assembly modifications that increase the infectivity of the progeny virions. Furthermore, the vesicular transport has been proven to accelerate release and spreading of the viral particles [41].

#### Rodent protoparvovirus-induced cell cycle arrest

The rodent protoparvoviruses MVM and H-1PV have the ability to induce cell cycle arrest in permissive cells - a process which is thought to favor viral replication [15,16,38,42]. The mechanisms underlying MVM-induced cell cycle arrest have been elucidated in part. During MVM infection, the NS1-mediated recruitment of cellular factors involved in viral DNA replication in APAR bodies leads to the shutoff of cellular genome replication and subsequent appearance of stalled replication forks [43]. As a consequence, MVM elicits a cellular DDR which is characterized by recruitment in the APAR bodies of components of the DNA damage machinery, such as H2AX, Nbs1, RPA32, Chk2, p53 and ATM [44]. This results in cell cycle arrest that, in the case of MVM, occurs in S/G2 phase, depending on the cell type and experimental conditions. While viral DNA and NS1 protein are required for the induction of the DDR, NS2 is dispensable, as infection of A9 cells with a MVM mutant lacking NS2 triggered a similar DDR response to the wild type (wt) virus [45]. Ectopic expression of NS1 was associated with increased levels of yH2AX phosphorylation, a DNA damage marker, although levels were much lower than those observed in virus-infected cells [44]. NS1 overexpression was also shown to induce cell cycle arrest but the mechanisms underlying this event may differ from those activated by the entire virus as the block in addition to S and G2, also occurs in G1 phase [15,38,46]. It has also been reported that the expression of NS1 is associated with single-strand DNA breaks, resulting in shut-down of cellular DNA replication [46].

The components of the DNA damage machinery which relocate into the APAR bodies are present in their active phosphorylated form. The ataxia telangiectasia mutated protein (ATM), a key regulator of the DNA damage checkpoint, is the kinase involved in the phosphorylation of these factors, as treatment with specific ATM inhibitors reduces phosphorylation [44]. Importantly, ATM-mediated DDR signaling participates in both the maintenance of MVM-induced cell cycle arrest and the regulation of virus multiplication, as inhibition of ATM activity affected both events [44]. Altogether these results provide evidence that cell cycle arrest is required for efficient virus multiplication. The importance of ATM signalling in MVM-induced cell cycle arrest was further corroborated by the finding that Chk2, a target of ATM phosphorylation, in MVM-infected cells, mediates the proteosomal degradation of CDC25A, a phosphatase required for cell cycle progression [47]. Moreover, MVM-induced cycle arrest is also characterized by cyclin B1 depletion, in a manner that is dependent upon the DDR response pathway [47]. As a consequence of DDR induction, p53 is also up-regulated in murine cells infected with MVM, and most likely contributes to the cell cycle arrest observed. Interestingly, despite p53 activation, protein levels of the cyclin kinase inhibitor (CKI) p21, a transcriptional target of p53, remain low during MVM infection. The virus itself induces p21 depletion through CRL4Cdt2 E3-ubiquitin ligase, which also re-localizes to MVM-APAR bodies. PCNA, a factor required for MVM-replication, functions as a scaffold protein for the p21/CRL4Ctd2 E3-ubiquitin ligase interaction and degradation [39]. Degradation of p21 is necessary for efficient virus replication, as the protein would otherwise negatively interfere with this process by interacting with and inhibiting PCNA and the CDK-2 [42]. These results demonstrate the ability of MVM to hijack, at multiple levels, the DDR and the cell cycle machinery to reshape the cellular milieu and redirect it towards a more efficient replication.

H-1PV also has the ability to induce a DDR, which results in a G2/M cell cycle arrest. However the underlying mechanisms are less well characterized than for MVM. We have described that virus infection is associated with accumulation of reactive oxygen species (ROS). ROS, which are a known source of DNA damage, contribute, at least in part, to both virus-induced DDR and cell cycle arrest, as antioxidant treatment protected the cells from these events [16]. Interestingly, the sole expression of NS1, similarly to the entire virus, is sufficient to trigger ROS accumulation, DNA damage and G2/M cell cycle arrest while, UV-irradiated virions, that lost the ability to express this protein, failed to alter cell cycle distribution [16]. These results indicate that NS1 plays a major role in H-1PV-induced cell cycle arrest. How exactly the protein induces ROS, however remains to be determined.

#### Rodent protoparvovirus-induced cell death

Cell cycle arrest leads to cell death and, eventually, to cell lysis. *Rodent protoparvoviruses* can kill cancer cells through different mechanisms. Virus-induced apoptosis has been reported in several cell lines [13,16,48-51], and is further supported by the fact that cellular regulators of both intrinsic and extrinsic apoptotic pathways are subjected to modulation upon parvovirus infection [10]. In addition to their role in regulating cell cycle arrest, ROS are important mediators of H-1PV-induced apoptosis, as antioxidant treatment, leading to a reduction of ROS levels, also decreased the fraction of cells undergoing apoptosis [16]. In glioma-derived cell lines, however, H-1PV induces apoptosis in only a small fraction of glioma cells and triggers instead an alternative cell death pathway involving the relocation of active cathepsins B and L from lysosomes into the cytosol and the downregulation of cystatin B and C levels, cystatin Band C (two cathepsin inhibitors) levels [52]. Interestingly, through this mechanism, H-1PV is able to circumvent the resistance of some glioma cells to apoptosis-inducing agents, thereby providing an alternative and efficient way to exert its oncolytic effect. Necrosis has also been observed as a consequence of RoPV infection [53,54]. As mentioned above, the non-structural viral protein NS1 plays a central role in the induction and regulation of the cytotoxic effects associated with parvovirus infection. The molecular mechanisms involved in NS1-mediated cell killing are still under investigation. However, some cellular partners and/or targets of NS1 have been identified, suggesting hypothetical pathways through which the parvoviral protein may induce cytotoxic effects. Protein kinase II (CKII) has been identified as an interaction partner of NS1 in MVM-infected cells, and it seems that NS1 cytotoxic activities correlate with the formation of a complex with CKII, since CKII knock-down is associated with cell resistance to virus oncolysis [55]. The NS1-CKII complex is involved in the phosphorylation of components of the cytoskeleton, in particular specific tropomyosin isotypes, leading to the rearrangement and degradation of tropomyosin filaments. This and other alterations of the cytoskeleton structure appear to contribute to virus-induced cytotoxicity [10].

For efficient parvoviral multiplication to take place, it is important that the death of the target cancer cell does not precede virus genome replication and production of progeny virions. The NS1-mediated activation of the cellular PDK1/PKB survival pathway may be one of the mechanisms evolved by the virus in order to delay host cell death [56].

Importantly, neither virus infection, nor the ectopic overexpression of the NS1 protein, is able to kill most normal cells [57,58]. This is believed to be related to a higher NS1 protein production in neoplastic cells, and to differences between normal and tumor cells in the occurrence of NS1 post-translational modifications that control NS1 activities. Some of the cellular factors mediating these modifications (for instance, PKC isoforms shown to stimulate the replicative and cytotoxic functions of NS1 through phosphorylation of specific NS1 residues) are generally up-regulated in cancer cells, which may sensitize these cells (compared with their normal counterparts) to virus-induced killing [10]. Therefore, the natural oncolytic activity of RoPV viruses can be traced back to the fact that cancer cells provide a more favorable intracellular milieu to both produce and activate viral cytotoxic effectors.

### *Rodent protoparvoviruses* as oncolytic agents

#### General features of oncolytic parvoviruses

Although RoPV viruses were frequently isolated from tumor cells, *in vitro* and *in vivo* studies have shown that these agents do not possess any tumorigenic activity but, on the contrary, exhibit the capacity to interfere with tumor growth [10,59]. Among RoPVs, the best characterized in terms of antineoplastic activity are the closely related MVM, LuIII and H-1PV which have the ability to infect various transformed cell lines of human origin. Several aspects of their biology make RoPVs particularly attractive agents for the development of anticancer strategies: (i) viral infection is asymptomatic and not associated with any disease in adults; (ii) there is a lack of pre-existing antiviral immunity in the human population; (iii) they display oncotropic, oncolytic and oncosuppressive properties, and (iv) they are able to elicit robust anticancer immune responses [59]. Indeed, MVM, LuIII and H-1PV are endowed with a natural preference to efficiently infect and kill tumor cells, while sparing normal cells. As described above, RoPVs can efficiently kill cancer cells by activating multiple cell death pathways, making unlikely the possibility of cancer cells acquiring resistance to viral cytotoxicity. On the contrary, there are examples in the literature describing an ability of the virus to overcome and efficiently kill cancer cells which are resistant to conventional and targeted therapy. Importantly, as will be discussed more extensively in the next paragraph, the virus, through lysis of the cancer cell, has the ability to elicit anticancer immune responses that contribute to the elimination of cancer cells, including those that were not directly infected by the virus.

RoPV oncoselectivity is not related to inefficient virus uptake by normal cells, but rather is due to the high dependence of the viral life cycle upon cellular factors that are dysregulated in tumor cells and involved in the control of cell proliferation and differentiation (see above).

However, the presence of cellular helper functions is not the only pre requisite that determines the permissiveness of target cancer cells for RoPV infection. Indeed, the inactivation of antiviral immune mechanisms may also contribute to the efficacy of virus infection. In mouse cells, MVM proved to be sensitive to type I interferon-mediated defence mechanisms [60]. Cancer cells are often deficient in their ability to mount this antiviral response, which may represent an additional factor in their sensitization to RoPV infection. Furthermore, it was shown that MVM is able to activate an antiviral cellular immune response in normal, but not in transformed mouse cells. While triggering the production of type I interferons (IFN-α/β) in normal mouse fibroblasts, thereby inducing an antiviral state, MVM activates an evading mechanism by blocking interferon synthesis in their transformed counterparts [60]. While RoPVs also fail to evoke a detectable IFN-β response in human cancer cells, the role of such an evasion process is presently disputed [61]. Besides enhanced production, the activity of the NS1 viral protein is also stimulated in transformed cells. For reasons yet unknown, NS1 exerts its cytotoxic effects in oncogene-transformed cells, but not in normal ones [62]. This qualitative modulation contributes additionally to the oncoselectivity of RoPV replication and lytic activity.

#### Activation of an anticancer immune response by oncolytic parvoviruses

As for other oncolytic viruses, the antineoplastic effects of RoPVs are due to direct oncolysis and/or to indirect immune reactions activated upon virus infection. These two events are not mutually exclusive, but most likely cooperate to achieve virus-mediated oncosuppression.

H-1PV infection of tumor cells stimulates the expression of danger- and pathogen-associated molecular patterns (DAMPs and PAMPs) and the release of tumor-associated antigens, thereby promoting the cross-presentation of tumor antigens by immature dendritic cells and facilitating the recognition of the tumor by the immune system [63]. The immunogenic features of RoPV-induced cell death were demonstrated in various systems including melanoma [64], glioma [63], and pancreatic cancer [65]. Furthermore, H-1PV was recently shown to induce pancreatic and colon carcinoma cells to display ligands to activating receptors of natural killer (NK) cells, resulting in enhanced NK cell-mediated killing of these cancer cells [66]. The immunostimulatory effect of RoPVs plays a significant role in their oncosuppressive activity. The occurrence of this immune component was evidenced by immunodepletion [67], immunoreconstitution [68] and immunostimulation [69] experiments. Further *in vivo* studies, performed in immunocompetent rats bearing two pancreatic tumors implanted at distant sites, showed that, when H-1PV was injected at only one tumor site, regression of the tumor located at the distal site could be observed. This bystander effect is believed to be mediated by the immune system as a result of its virus-dependent activation at the injected site, in keeping with the accumulation of granzymes and perforin at the distal tumor site [59]. The immune component of RoPV oncosuppression was also demonstrated through adoptive transfer of immune cells. Splenocytes transferred from immunocompetent rats bearing H-1PV-treated orthotopic pancreatic tumors to rats bearing the same, but uninfected, tumors were found to protect the recipient animals from developing cancer, providing evidence that activation of an antitumor immune response occurs upon H-1PV infection [70]. The induction of an antitumor immune response by H-1PV correlates with both stimulation of IFN-γ production, and increase in the T helper cell population, as demonstrated in splenocytes from infected tumor-bearing animals, as well as in human peripheral blood mononuclear cells (PBMC) infected *in vitro* [60]. Altogether, these results provide strong evidence that H-1PV treatment triggers an antitumor immune response contributing to the success of cancer virotherapy.

On the other hand, an antiviral humoral immune response, and more specifically a production of virus-neutralizing antibodies, was detected in animal models within one week after infection with H-1PV, MVM or the Mouse parvovirus MPV-1 [67,71,72]. Neutralizing antibodies are expected to impede virus propagation and ensuing oncosuppression. It is noteworthy in the context of clinical applications of RoPVs, that no or little seropositiveness to these agents can be detected in the human population [73], providing a window between treatment and seroconversion for unimpeded viral activity. The capacity of RoPVs to elicit antiviral cellular immune responses is less clear [71] but deserves consideration. However, the interference of these cellular antiviral responses with tumor suppression is not necessarily negative and may actually play a key role in the efficacy of oncolytic virotherapy. For example, in an intracranial murine model of metastatic melanoma, the virus-specific cytotoxic lymphocyte response was shown to be critical to neuroattenuated HSV-mediated tumor suppression [74]. Similarly, in a B16 melanoma model, the efficiency of vesicular stomatitis virus (VSV)-induced oncolysis was strongly dependent on virus-specific NK and CD8 T cells [75].

#### Preclinical studies supporting the use of oncolytic parvoviruses as anticancer agents

In this paragraph, special emphasis will be given to H-1PV that has been evaluated extensively at the preclinical level as an anticancer agent using a number of different *in vitro* and *in vivo* tumor models, as summarized in Table 1.

**Table 1:** **Antineoplastic effects of H-1PV on different human tumor entities**

**Tumor entity**	***In vitro*** **system**	***In vitro*** **effect**	***In vivo*** **system**	***In vitro*** **effect**	**References**
**Glioblastoma and gliosarcoma**	Cell lines and primary cells	Dose-dependent killing at low MOIs	Immunocompetent Wistar rats; Nude rats	Complete tumor regression and increased animal survival	[77,79]
**Neuroblastoma**	Cell lines	Highly efficient cell killing	N.D.	N.D.	[48]
**Medulloblastoma**	Cell lines	40-100% cell lysis	N.D.	N.D.	[84]
**Burkitt’s lymphoma**	EBV positive and negative cell lines	80-100% cell death	SCID mice	Tumor regression and significant prolongation of animal survival	[85]
**Breast**	Cell lines and primary cells	20-80% cell death	Nude mice	Inhibition of tumor formation; tumor regression	[86,88,89]
**Pancreatic carcinoma**	Cell lines and short-term cultures	20-100% cell killing	SCID mice; Nude rats	Tumor regression	[13,91]
**Cervical carcinoma**	Cell lines	40-100% cell lysis	SCID mice; Nude rats	Tumor regression	[13,96]
**Gastric tumors**	Cell lines	20-80% cell death	Nude mice	Inhibition of tumor formation	[98]
**Melanoma**	Cell lines	Cell killing	N.D.	N.D.	[64]
**Hepatoma**	Cell lines	80% reduction of metabolic activity	Nude mice	Inhibition of tumor formation	[99]
**Colon**	Cell lines	Limited virus replication and cytotoxicity	N.D.	N.D.	[66,102]

N.D. Not determined; EBV, Epstein-Barr virus.

(i) Glioma
  Glioblastoma multiforme (GBM) is the most common and aggressive type of primary brain tumor in humans, for which the median survival is 15 months after initial diagnosis [76]. New therapeutic approaches are urgently needed since the current treatments, represented by surgery, chemo- and radio-therapy, are not effective against this type of tumor. Glioblastoma and gliosarcoma-derived cell lines were shown to be permissive for H-1PV replication and gene expression and to be highly susceptible to H-1PV-induced cytotoxicity [77]. The efficiency of H-1PV-mediated killing of cultured human glioma cells prompted investigations of the anti-glioma effects of this virus *in vivo*. Upon intratumoral or intravenous delivery into rat orthotopic models of rat or human gliomas, H-1PV was able to induce complete tumor regression and to increase animal survival without causing inflammation or alteration of surrounding normal tissues [78]. Virus infection was associated with cathepsin B induction in the tumor but not in normal tissues, substantiating *in vitro* evidence that H-1PV induces a lysosomal type of glioma cell death [79]. Intranasal administration of H-1PV is also an efficient route of virus delivery in the case of brain tumors, as demonstrated by viral replication and spreading at the tumor site [80].
  Interestingly, a combination approach using ionizing radiation and H-1PV showed increased cytotoxicity in a set of glioma-derived cell lines, including radio-resistant cells, compared to treatment with single agents. This effect could be observed more particularly when infection was performed after irradiation, and was associated with an increase in both NS1 levels and S-phase cell fraction. Importantly, this study demonstrated that previous exposure of glioma cells to radiation does not affect their susceptibility to H-1PV infection, supporting the potential clinical use of H-1PV in pre-irradiated glioma patients [78]. Recently, twelve different *Protoparvovirus* members, including H-1PV, LuIII and MVM, were compared for their efficacy in killing a panel of glioma-derived cell lines. LuIII and H-1PV showed the greatest oncolytic activity when cells were infected with high multiplicities of infection (MOIs), while LuIII displayed superior anticancer activity when viruses were used at low concentrations, with an ability to propagate efficiently in all glioma cell lines tested. Interestingly, LuIII reduced tumor growth in mouse xenograft models when administrated both intratumorally and systemically, with no apparent adverse side effects for the animals [81].
(ii) Neuroectodermal tumors
  H-1PV oncolysis has also been reported in paediatric neuroectodermal tumors, such as neuroblastoma and medulloblastoma. Neuroblastoma is a highly malignant solid tumor originating from the central nervous system and is characterized by N-myc gene amplification, which is a biomarker of poor prognosis. Screening 11 neuroblastoma-derived cell lines for H-1PV sensitivity showed oncolytic effects in all the lines tested, irrespective of their p53 mutation or N-myc amplification status. Cytotoxic effects were associated with efficient viral replication, gene expression, production of progeny virions and induction of cell cycle arrest and apoptosis [48]. Interestingly, neuroblastoma-derived cell lines were especially susceptible to H-1PV oncolysis, supporting the concept of using H-1PV as an anticancer agent against this tumor entity.
  Medulloblastoma is the most common malignant brain tumor in children, while in adults its occurrence is rare. The potential of oncolytic virotherapy for the treatment of medulloblastoma is supported by the ability of several oncolytic viruses (Seneca Valley virus, reovirus and recombinant measles virus) to efficiently target this tumor type [82,83]. In a recent study, H-1PV was shown to be cytotoxic for a range of medulloblastoma-derived cell lines. Interestingly, virus-induced repression of genes which are involved in the maintenance of the neural progenitor state (including the oncogene myc), and whose amplification is associated with malignant transformation and poor prognosis, may have contributed to the oncolytic effect observed [84].
(iii) Lymphoma
  Burkitt’s lymphoma is a sporadic form of lymphoma, which is often associated with Epstein-Barr virus (EBV) infection. Although current treatment options led to improved survival in adults, an efficient cure is not yet available and, as a result, innovative treatments are needed. In this regard, H-1PV may be a potential candidate since *in vitro* and *in vivo* studies have demonstrated the ability of the virus to efficiently kill Burkitt’s lymphoma-derived cell lines (most likely through a necrotic mechanism and irrespective of EBV positivity) and to induce complete tumor destruction in severe combined immunodeficient (SCID) mice, accompanied by viral replication and spreading [85]. Furthermore, diffuse large B cell lymphoma (DLBCL), cutaneous T cell lymphoma (Cezary syndrome), large cell immunoblastic lymphoma, T cell acute lymphoblastic leukemia (T-ALL), and acute promyelocytic leukemia cells were killed efficiently by H-1PV *in vitro*; interestingly, oncolytic virus infection of these cells was productive in most cases (Angelova et al, unpublished observations).
(iv) Melanoma
  Killing effects have been observed in human melanoma-derived cell lines infected with H-1PV, indicating that the virus may also be able to target this tumor type. H-1PV-induced melanoma cell death was found to be immunogenic, leading to the activation of antigen-presenting cells and tumor-specific cytotoxic T lymphocytes [64].
(v) Mammary carcinoma
  Breast cancer is another potential tumor targetable by H-1PV, as shown both *in vitro* and in a xenograft mouse model [86,87]. Furthermore, short-term cultures of patient-derived breast cancer cells were shown to be highly sensitive to the cytotoxic effects induced by the virus [86-89].
(vi) Pancreatic carcinoma
  A number of studies have investigated the therapeutic potential of H-1PV against pancreatic ductal adenocarcinoma, a cancer type classified among the most frequent cause of cancer-related deaths, and for which effective treatment options are still lacking. Gemcitabine is a chemotherapeutic drug currently in use for the treatment of advanced local and metastatic pancreatic cancer. Unfortunately, resistance of cancer cells to gemcitabine dramatically reduces the efficacy of the therapy and causes tumor recurrence [90]. H-1PV cytotoxic effects have been reported in pancreatic cancer-derived cell lines and in animal tumor models, without being associated with significant deleterious side effects [91]. Interestingly, H-1PV could kill both gemcitabine-sensitive and resistant cell lines. Moreover, the oncosuppressive effects of H-1PV could be further enhanced by combining the virus with gemcitabine, suggesting an interesting novel combination option for the treatment of pancreatic carcinomas. The susceptibility of pancreatic cancer-derived cell lines to H-1PV infection was reported to correlate with the expression of functional SMAD4, a transcription factor which is involved in the regulation of the tumor growth factor β (TGF-β) signaling pathway [92], and whose gene is mutated in about 50% of pancreatic carcinomas. Mutations or deletions of SMAD4 have been associated with a decrease in the sensitivity of pancreatic cancer cells to H-1PV, which was traced back to the involvement of SMAD4 in parvovirus P4 promoter activation and ensuing NS1 expression [93]. Furthermore, as mentioned previously, H-1PV exerts immunostimulatory effects through the infection of pancreatic carcinoma-derived cells. Although pancreatic cancer generally exhibits low immunogenicity, it was shown that pre-treatment of NK cells with interleukines stimulated NK cell recognition of tumor cells. H-1PV infection of pancreatic cancer cells further sensitized these cells to NK cell-mediated killing by stimulating the release of cytokines and chemokines known to recruit immune cells to the tumor site, and by increasing the expression of ligands to NK cell-activating receptors on the cancer cell surface [94]. In a recent study [68], the release of high-mobility group box protein B1 (HMGB1) from H-1PV-infected pancreatic ductal adenocarcinoma (PDAC) cells was reported, providing further evidence of the immunogenic nature of H-1PV-induced PDAC cell death. Importantly, this virus-mediated danger signaling process was reinforced by standard anti-PDAC cytotoxic treatments (gemcitabine), supporting the concept of H-1PV inclusion in multimodal treatments for pancreatic cancer patients. Furthermore, co-injection of recombinant IFN-γ was shown in an orthotropic pancreatic carcinoma model to enhance activation of macrophages and splenocytes, virus spreading to metastatic sites and animal survival, warranting clinical investigation of this promising strategy [69].
(vii) Cervical carcinoma
  Cervical cancer is the second most common cancer in women. Although prevention screenings have significantly reduced mortality, and the vaccination program against human papillomaviruses, the major cause of cervical cancer, is expected to reduce tumor incidence among young women, the prognosis for the advanced disease remains poor, with a 1-year survival of only 15-20% [95]. H-1PV is a promising therapeutic agent for the treatment of cervical carcinoma. Indeed, it has been reported that H-1PV infection induces tumor regression in SCID mice implanted with the human cervical cancer-derived HeLa cells, in a dose-dependent manner and in correlation with viral gene expression [96]. Interestingly, our group recently showed that H-1PV efficacy against cervical carcinomas can be potentiated by combining the virus with histone deacetylase (HDAC) inhibitors [13]. In particular, sublethal doses of valproic acid (VPA) synergistically enhanced H-1PV-induced killing effects in cervical carcinoma cell lines. Remarkably, in a HeLa xenograft rat model, H-1PV/VPA co-treatment induced complete tumor regression without causing adverse side effects at doses of virus that were ineffective as a monotherapy. This H-1PV/VPA cooperative effect was shown to be associated, both *in vitro* and *in vivo,* with increased induction of oxidative stress, DNA damage and apoptosis, as well as with enhanced viral DNA replication, gene expression and production. The efficacy of the H-1PV/VPA combination was also demonstrated in a range of pancreatic cancer-derived cell lines and rodent xenograft models of human pancreatic carcinomas, indicating that this combination may represent a promising approach also for the treatment of this type of cancer.
(viii) Gastric carcinoma
  H-1PV-induced killing of human gastric cancer cells was found to follow the up- or downregulation of the expression of a number of cellular genes [97]. Recombinant NS1 expression was also shown to reduce the tumorigenic potential of poorly differentiated gastric cancer cells implanted in nude mice, providing evidence that this viral protein interferes with the growth of these cells [98]. These studies warrant further testing of the antineoplastic effects of H-1PV on gastric tumors.
(ix) Hepatoma
  H-1PV oncolytic effects have been reported in hepatoma-derived cancer cell lines, in which virus infection was associated with viral DNA replication, gene expression and virus production. Importantly, no cytotoxicity was observed in H-1PV-infected normal primary hepatocytes [99]. Upon infection with H-1PV, sensitive human hepatoma cells showed a global repression of genes involved in cell proliferation, growth and apoptosis [100]. *Ex vivo* or *in vivo* infection with H-1PV also inhibited tumor development from human hepatoma xenografts in nude mice [101].
(x) Colon carcinoma
  In comparison with the tumor types described above, human colon cancer appears to exhibit lower susceptibility to the oncolytic activity of H-1PV, as suggested by the fact that infected colon carcinoma-derived cell lines show little cytopathic effects and fail to sustain efficient viral replication [102]. However, it is noteworthy that H-1PV infection of colon cancer cells potentiates their NK cell-mediated killing by up-modulating the expression of NK cell-activating receptor ligands displayed on the tumor cells [66]. Therefore, although inefficient at inducing a direct oncolytic effect in colon carcinoma, H-1PV may still be of therapeutic value against this tumor by enhancing the ability of infected colon carcinoma cells to activate innate immune cells.

#### ParvOryx 01: the first clinical trial using an oncolytic parvovirus

The preclinical evidence obtained in glioma cultures and animal models provided the basis for the launching of a first phase I/IIa clinical trial, named ParvOryx01, involving patients with GBM.

Besides the above-mentioned preclinical evidence of H-1PV anti-glioma effects, the ability of H-1PV to cross the blood-brain barrier and infect intracerebral tumors after systemic administration to rats gives hope for the development of non-invasive, H-1PV-based treatments of GBM [103]. The trial started in late 2011 and involves 18 adult patients diagnosed with GBM grade IV, recurring in spite of chemo- and radio therapy. This is a phase I/IIa dose escalation study aimed primarily at confirming H-1PV safety, and secondarily at gathering preliminary evidence of anticancer efficacy. The study design comprises two arms differing in the first step of the treatment, namely intratumoral versus intravenous administration of half of the total virus dose, and sharing the second step of the treatment, namely tumor resection at day 10 and administration of the remaining half of the total virus dose into the wall of the resection cavity [76]. The trial is still in progress, and no clinical conclusions can be drawn at the present time besides safety of H-1PV up to a dose of 10^9^ infectious units per patient. Biological monitoring is conducted by analysing the patients’ bodily fluids and resected tumor. The results of this accompanying research are intriguing, showing that H-1PV achieves widespread infection of gliomas, also after intravenous administration, and undergoes at least limited replication and expression in neoplastic tissues. Interestingly, this virus replication in some tumors is associated with necrosis and massive infiltration with T cells, including CTLs, some of which appear to be specific for viral and glioma epitopes (unpublished results). We trust that the further analysis of these surrogate markers of efficiency, as well as clinical data, will warrant the future extension of parvovirus clinical studies to subsequent phases, including also second-generation RoPV-based (combination) treatments.

### Developing novel RoPV-based therapies with enhanced anticancer efficacy

In recent years, H-1PV has been the subject of genetic manipulations that aimed at increasing virus oncospecificity and anticancer efficacy in order to optimize the therapeutic potential of RoPV-based treatments. Genetic engineering of the H-1PV capsid proved to be a suitable approach to increase virus specificity for cancer cells at the level of cell recognition and entry. The above-mentioned determinants of RoPV oncotropism indeed operate at the intracellular level. In consequence, the wild-type virus is able to enter most normal cells, which undergo a cryptic or abortive infection that is clinically unapparent but results in the sequestration and loss of a major fraction of inoculated virions away from the tumor target. In a first step, sites were identified on the capsid, which were involved in binding to normal cellular receptors, and whose modifications led to virus detargeting, i.e. lack of uptake by normal cells. In a second step, a site was identified within the viral capsid tolerating the insertion of the RGD-4C cyclic peptide. This peptide recognizes α_v_β_3_ and α_v_β_5_ integrins, which are typically overexpressed on cancer cells and angiogenic blood vessels, and was used as a model ligand for virus retargeting to these cells. Indeed, the RGD-4C-decorated virus showed increased specificity for cancer cells, while retaining the oncolytic potential of the parental virus [28]. This study provided proof-of-concept demonstration of the possibility of minimizing parvoviral entry into normal cells for the benefit of treatment safety and efficacy, and paved the way for further retargeting either by grafting other peptidic ligands of cancer-specific receptors (such as the epidermal growth factor receptor overexpressed in gliomas [104]) or by inserting peptide libraries to be used for *in vitro* and *in vivo* screenings.

In another approach, the H-1PV genome was inserted into a replication-defective adenovirus vector genome, with the aim of generating an adenovirus-parvovirus chimera. The chimeric vector multiplied to high-titers, thus allowing the large-scale production of parvoviral DNA-containing particles, which is generally difficult to achieve with current parvovirus production procedures, rendering the chimera virus more suitable for clinical applications. The parvoviral genome was efficiently released from the adenoviral DNA backbone in target cancer cells, leading to autonomous H-1PV replication, cytotoxic effects and production of fully infectious parvoviral particles. The adenoviral carrier allowed H-1PV to be delivered into tumor cells originally resistant to direct parvovirus entry, and to kill these cells under conditions in which neither parental virus was efficient. While showing this broader oncolytic activity, the adeno/parvoviral chimera kept the innocuousness of its infectious parvovirus component for normal cells, thereby warranting further (pre)clinical assessment *in vivo* [105]. Future studies will be directed toward the development of a second generation of Ad-PV chimeras with improved anticancer activity either by arming the chimera with proapoptotic or immunostimulatory transgenes, or by improving its cancer specificity by using retargeted Ad as a backbone.

Interestingly, the immunomodulatory effect of H-1PV could be enhanced by arming the virus with specific PAMPs, namely CpG motifs [106]. It remains to be explored whether other (immuno) regulatory elements could be introduced into the RoPV genome, considering the fact that the virus, owing to its limited packaging capacity, does not tolerate the insertion of transgenes larger than a few hundred nucleotides for infectivity to be retained.

It has been observed that cancer cell lines differ in their sensitivity to RoPV infection, with some of them being poorly permissive to virus replication and cytotoxicity. RoPV replication in human cancer cells can be enhanced through virus adaptation. Isolation of virus variants adapted for efficient production and spreading in human glioma cell cultures was recently achieved by serial passaging H-1PV in semi-permissive glioma cells (Nuesch et al. unpublished results).

As mentioned above, combinations of H-1PV with physical (ionizing radiation) and chemical (gemcitabine, temozolomide) cytotoxic agents, or with epigenetic modulators (HDAC inhibitors) resulted in striking synergistic oncolytic effects both in cell culture and animal models, providing the preclinical proofs of concept needed to move these protocols into the clinic. We anticipate that future studies will be directed toward the identification of other anticancer agents or therapeutic modalities (e.g. immunotherapy) that may act additively or synergistically with the virus in eliminating cancer cells, reinforcing the antineoplastic activity of the virus whilst keeping its excellent safety profile (see also next paragraph). This advance may also allow a reduction of the therapeutic dose to be used in cancer patients while maintaining or even enhancing efficacy.

### Future challenges

Oncolytic viruses are gaining momentum as a novel form of anti-cancer therapy. As demonstrated in the numerous pre-clinical models described above, RoPVs display striking oncosuppressive effects whilst being non-pathogenic to humans. For the full potential of these anti-cancer agents to be exploited, we believe that the development of more complex treatment strategies, aimed at enhancing virus replication, direct oncolytic activity and/or immunological adjuvant effects, will be necessary. Indeed, the varying susceptibility of tumor cell lines to RoPV replication and cell killing observed *in vitro* indicates that further optimisation of RoPV-based therapy is likely to be critical for the treatment of advanced human cancers characterised by high intratumoral heterogeneity and genomic instability.

#### Combination therapies

To this end, we anticipate that combination therapy using agents that synergize with RoPVs, may hold the key for maximizing anti-cancer effects. The permissiveness of a given cancer cell for RoPV infection and replication is governed by an intricate set of virus/host interactions which, as is the case with many OVs, remain largely unknown. A more thorough understanding of the parvoviral life-cycle and host factors implicated in it may reveal novel targets for therapeutic intervention, thus guiding the development of RoPV-based combination therapies with augmented anti-neoplastic activity. The power of such rationally designed combinations to reinforce RoPV therapy is exemplified by the synergism observed between H-1PV and HDAC inhibitors, following the discovery that acetylation of NS1 enhances its cellular functions [13]. To efficiently dissect the relationship between RoPVs and the host, one particularly promising strategy is the high-throughput RNAi screening (htRNAi), a platform which facilitates genome-wide analysis of the cellular factors involved in virus life-cycles [107]. When used for the first time to identify host factors that modulate Maraba virus-induced oncolysis, htRNAi revealed an unexpected synergistic target (the ER stress response) for OV therapy, demonstrating the capacity of this technology to direct effective combination treatments [108].

The above-mentioned rational design of combination therapy requires prior knowledge of particular virus host interactions. As an alternative approach, high-throughput screening of drug and chemical libraries may be performed in an unbiased fashion to identify compounds that potentiate OV therapy. This approach has already shown promise as an efficient way to discover novel enhancers of OV activity. For example, equilibrative nucleoside transporter-1 (ENT1) antagonists were found to be potent amplifiers of oHSV replication following a drug screening to identify molecules that increased viral spread [109], whilst a chemical library screen for compounds that sensitise cancer cells to viral cytotoxicity revealed a previously unidentified small molecule as a synergistic enhancer of VSV-mediated cell killing [110].

#### More relevant disease models for proof-of-concept validation

Following the identification of promising PV-drug combinations *in vitro,* their safety and efficacy must be corroborated *in vivo*. Pre clinical validation of RoPV-based therapies is traditionally performed using two classes of *in vivo* systems: (i) xenograft models, generated from the implantation of cultured human tumor cells into immunocompromised rodents, and (ii) immunocompetent syngeneic models, bearing tumors originating from the same species. Despite providing valuable indications of efficacy, both approaches carry significant drawbacks that limit their utility as predictive tools of clinical outcome: xenograft models will not reveal the positive or negative consequences of the host immune response to treatment, whilst results from syngeneic animal models can be misleading since the behavior of the virus within cells, as well as immune response towards it, may vary considerably between species. Furthermore, tumors established from cultured cell lines seldom recapitulate the complexity or heterogeneity of those observed in patients. For predicting the clinical outcome of novel RoPV-based treatments and selecting the best combination therapies to move towards the clinic, patient-derived tumor xenograft models are likely to be more informative as they retain greater similarity to parental tumors [111]. Considering the anticipated importance of an immune-mediated component of therapy, the use of novel ‘humanised’ animal models may also be of particular value for pre clinical RoPV validation, as evidenced by a recent study of H-1PV oncolytic and immunostimulating effects against PDAC [68]. Reconstituted animals develop a functional human immune system whilst permitting the engraftment of human tumor cells [112], making them powerful tools for studying the immune response during cancer therapy [113].

#### Upscaling of virus production

Another significant challenge facing the field, which may hamper the successful translation of these therapeutics to the clinic, is the large-scale production of RoPV-based vectors in compliance with Good Manufacturing Practice (GMP). To produce RoPV vectors at the quality and quantity required to meet clinical demand, major efforts must be directed towards the search for more productive packaging cell lines and optimisation of culture media and growth conditions, as well as the improvement of purification procedures. The development of high-yielding, scalable bioreactor platforms using cell lines growing in suspension is also an important goal for reducing the considerable costs of virus production.

#### Patient stratification

**Figure 3:**
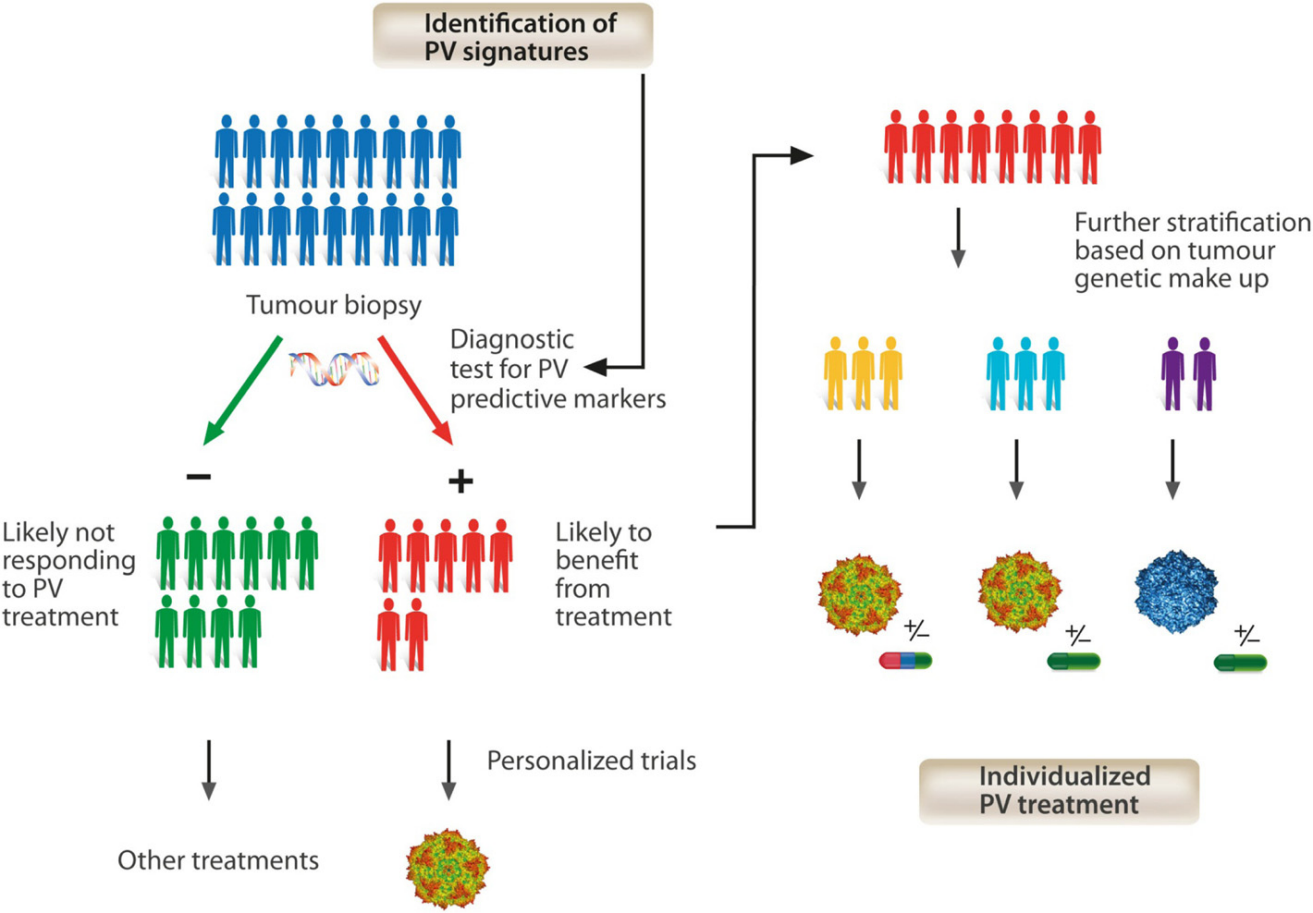
**Towards individualized parvovirus-based treatments.** A better understanding of PV-host interaction with identification of key cellular factors playing a role in the PV-life cycle and in virus-mediated cytotoxicity may provide valuable hints for a more rational and efficient use of PV-based therapies in clinical settings. Predictive tests may identify patients with a molecular portrait that makes them more likely to benefit from virus treatment or targeted combination therapies.

Looking forward, we believe that the identification of biomarkers predictive of sensitivity/resistance to RoPV therapy will also be a critical step towards its effective use in clinical settings. With this knowledge, patients may be screened for the presence of specific markers and selected for “smart” clinical trials based upon their likelihood of responding to treatment. Moreover, in those patient subsets exhibiting certain features that could conceivably compromise efficacy, outcome may be improved through the rational application of combination therapy and/or the use of engineered RoPVs. Figure 3 summarises the concept that patients are stratified and RoPV-based treatment regimens customised to individual needs.

#### Earlier-line virotherapy

The successful translation of laboratory results into effective cancer treatments requires more than just talented researchers and inspired clinicians. Early-phase trials of oncolytic viruses are typically conducted on patients with advanced or recurrent cancers, for whom all other therapeutic options have failed. However, conventional first-line treatments of some forms of cancer, including glioblastoma multiforme, provide only modest benefit to the patients and are often associated with severe adverse side-effects. Furthermore, the ability of patients to mount a robust immune response is often compromised at such late stages of disease, which may prevent them from benefiting from the immunostimulating action of RoPVs. Long-term therapeutic improvements may therefore be gained primarily by patients treated at early stages of their disease. This raises a delicate regulatory issue: should patients with aggressive cancers, for which no curative therapies are currently available, have access to novel treatments at an earlier point after diagnosis? While lying beyond the scope of this review, this regulatory issue deserves more discussion.

Exciting results at the pre clinical level offer a tantalizing glimpse of the potential of oncolytic parvoviruses as novel anti-cancer agents. However, the successful translation of these promising laboratory results into clinically effective treatments is still in its infancy. We anticipate that the ongoing clinical trial will provide valuable information enabling the further exploitation of RoPV therapeutic properties, as well as revealing hurdles that may limit efficacy. Overcoming these limitations represents a challenge to be taken up first at the bench level. Guided by better understanding of the cellular factors controlling RoPV-host interactions and malignant progression, we endeavor to develop next-generation RoPV-based treatments with improved anticancer efficacy. This effort will hopefully contribute to reinforce the impact of RoPVs on the fight against this devastating disease.

## Conclusions

Oncolytic virotherapy of cancer has emerged as a promising alternative to toxic chemotherapy regimens. Among a dozen oncolytic viruses presently tested at the clinical level, rodent parvoviruses (and the H-1 parvovirus in particular) attract considerable attention, due to their remarkable natural oncoselectivity and lack of pathogenicity for humans. In the last two decades, an impressive amount of preclinical data has been accumulated, showing that H-1PV possesses both oncolytic and immunostimulating properties. These give the virus a significant cancer therapeutic potential, either alone or in combination with other agents (e.g. histone deacetylase inhibitors), as revealed in several tumor models. Cancers targeted by RoPVs include glioblastoma multiforme, the most common and aggressive primary brain tumor in humans. The first phase I/IIa clinical trial using an oncolytic parvovirus (H-1PV) was launched in 2011 for patients with recurrent GBM. We trust that this ongoing clinical study will pave the way for subsequent efficacy trials, and prompt the development of next-generation parvovirus-based anticancer therapeutics.

## Abbreviations

OVs: Oncolytic viruses
HSV: Herpes simplex virus
RoPV: Rodent protoparvovirus
KRV: Kilham Rat Virus
MPV: Mouse Parvoviruses
MVM: Minute Viruses of Mice
PKC: Protein kinase C
CRM: Chromosome region maintenance
SAT: Small alternatively translated
ER: Endoplasmic reticulum
MHC: Major histocompatibility complex
PLA2: Phospholipase A2
NPC: Nuclear pore complex
NLS: Nuclear localization signals
CDK-1: Cyclin-dependent kinase-1
APAR: Autonomous parvovirus-associated replication
RPA: Replication protein A
PCNA: Proliferating cell nuclear antigen
DDR: DNA damage response
ATM: Ataxia telangiectasia mutated
CKI: Cyclin kinase inhibitor
ROS: Reactive oxygen species
IFN: Interferon
DAMPs: Danger-associated molecular patterns
PAMPs: Pathogen-associated molecular patterns
NK: Natural killer
VSV: Vesicular stomatitis virus
GBM: Glioblastoma multiforme
MOI: Multiplicity of infection
EBV: Epstein-Barr virus
SCID: Severe combined immunodeficient
DLBCL: Diffuse large B cell lymphoma
T-ALL: T cell acute lymphoblastic leukemia
HMGB1: High-mobility group box protein 1
PDAC: Pancreatic ductal adenocarcinoma
HDAC: Histone deacetylase
VPA: Valproic acid
GMP: Good Manufacturing Practice
